# Prevalence of burnout syndrome in intensivist doctors in five Brazilian capitals

**DOI:** 10.5935/0103-507X.20160053

**Published:** 2016

**Authors:** Márcia Oliveira Staffa Tironi, José Mário Meira Teles, Dalton de Souza Barros, Débora Feijó Villas Bôas Vieira, Colbert Martins da Silva Filho, Davi Felix Martins Júnior, Marcos Almeida Matos, Carlito Lopes Nascimento Sobrinho

**Affiliations:** 1 Escola Bahiana de Medicina e Saúde Pública - Salvador (BA), Brazil.; 2 Instituto de Gestão em Saúde, Hospital de Urgências de Goiânia - Goiânia (GO), Brazil.; 3 Hospital das Clínicas, Faculdade de Medicina, Universidade São Paulo - São Paulo (SP), Brazil.; 4 Universidade Federal do Rio Grande do Sul - Porto Alegre (RS), Brazil.; 5 Universidade Estadual de Feira de Santana - Feira de Santana (BA), Brazil.

**Keywords:** Burnout, professional/epidemiology, Working conditions, Physicians/psychology, Occupational diseases/epidemiology, Prevalence, Intensive care units, Intensive care units, pediatric, Intensive care, neonatal

## Abstract

**Objective:**

To estimate the prevalence of burnout in intensivist doctors working in adult, pediatric and neonatal intensive care units in five Brazilian capitals.

**Methods:**

Descriptive epidemiological study with a random sample stratified by conglomerate with 180 intensivist doctors from five capitals representing the Brazilian geographic regions: Porto Alegre (RS), Sao Paulo (SP), Salvador (BA), Goiania (GO) and Belem (PA). A self-administered questionnaire examining sociodemographic data and the level of burnout was evaluated through the Maslach Burnout Inventory.

**Results:**

A total of 180 doctors were evaluated, of which 54.4% were female. The average age was 39 ± 8.1 years, 63.4% had specialization as the highest degree, 55.7% had up to 10 years of work experience in an intensive care unit, and 46.1% had the title intensive care specialist. Most (50.3%) had weekly workloads between 49 and 72 hours, and the most frequent employee type was salaried. High levels of emotional exhaustion, depersonalization and inefficacy were found (50.6%, 26.1% and 15.0%, respectively). The prevalence of burnout was 61.7% when considering a high level in at least one dimension and 5% with a high level in three dimensions simultaneously.

**Conclusion:**

A high prevalence of burnout syndrome among intensivist doctors was observed. Strategies for the promotion and protection of health in these workers must be discussed and implemented in hospitals.

## Abstract (pt)

**Objetivo:**

Estimar a prevalência de *burnout* em médicos intensivistas que trabalham em unidades de terapia intensiva adulto, pediátrica e neonatal, de cinco capitais brasileiras.

**Métodos:**

Estudo epidemiológico descritivo, com amostra aleatória e estratificada por conglomerado, de 180 médicos intensivistas de cinco capitais, representando as regiões geográficas brasileiras: Porto Alegre (RS), São Paulo (SP), Salvador (BA), Goiânia (GO) e Belém (PA). Um questionário autoaplicável avaliou dados sociodemográficos e o nível de *burnout* foi avaliado por meio do *Maslach Burnout Inventory*.

**Resultados:**

Foram avaliados 180 médicos, sendo 54,4% do sexo feminino. A média de idade foi 39 ± 8,1 anos, 63,4% com a especialização como a maior titulação, 55,7% com até 10 anos de trabalho em unidade de terapia intensiva e 46,1% possuíam título de especialista em terapia intensiva. A maioria (50,3%) tinha carga horária semanal de trabalho entre 49 e 72 horas, e o tipo de vínculo mais frequente foi empregado assalariado. Níveis elevados de exaustão emocional, despersonalização e ineficácia foram encontrados em 50,6%, 26,1% e 15,0%, respectivamente. A prevalência de *burnout* foi de 61,7%, quando considerado nível alto em pelo menos uma dimensão e de 5% com nível alto nas três dimensões simultaneamente.

**Conclusão:**

Observou-se elevada prevalência da síndrome de *burnout* entre os médicos intensivistas. Estratégias para promoção e proteção à saúde desses trabalhadores devem ser discutidas e implementadas nos hospitais.

## INTRODUCTION

Daily work in intensive care settings requires the intensivist to develop skills that allow him/her to manage demands inherent to two poles of performance (the technical/scientific and the relational). The former, which is more objective, imposes continuous scientific updating to obtain advanced knowledge in this area, whereas the latter, which is more subjective, demands the development of sensitivity, which should allow the individuals to recognize the needs of the human beings who are the subject/object of their work. Tironi et al.^(1)^ conducted a study with intensivists that demonstrated a high prevalence of burnout syndrome among these professionals and demonstrated that in addition to improving the quality of care provided, the balance between the two poles can be a factor associated with health protection in these workers.

Burnout is a psychological syndrome involving professional exhaustion that arises from chronic emotional overload in work that involves interpersonal relationships with great responsibility. Burnout presents three interdependent dimensions: emotional exhaustion, depersonalization and inefficacy.^(2-6)^ Exhaustion represents the individual component, with feelings that an individual is required to perform beyond his/her resources. Depersonalization refers to the interpersonal component and, at high levels, can give an initial impression of defensiveness and protection with a risk of chronic detachment. Inefficacy is the self-assessment component and is typically accompanied by feelings of incompetence and low productivity.^(3,7)^

Burnout is related to the provision of services. Vulnerability to its development increases when this interaction involves a significant load of responsibility, protection and care for another, which occurs in intensivist work.^(2,7)^ It is also important to recognize that burnout must be understood as a process with a close relationship with the context in which this service is provided, which helps to shape this phenomenon. Many authors^(3-5)^ highlight that workers suffer more due to issues linked to the context of work than individual characteristics.

Considering the importance of intensivists for the care of gravely sick people and the possibility that the repercussions of burnout can lead to an inability to work, which can compromise patient care, the early identification of this syndrome can assist with interventions (individual and/or organizational) for the prevention of these situations.^(7,8)^ Some Brazilian^(1,9)^ and international^(10-14)^ studies have made efforts to better understand the issue. However, the majority of these studies did not address the full complexity of the work in the intensive care unit (ICU). Issues with the sample size, considering a single hospital/city, or even studying only a single ICU type (adult, neonatal or pediatric) resulted in these studies leaving a gap in the knowledge on this topic. Without the pretense of fully covering the topic but with the purpose of contributing significantly, our study aims to estimate the prevalence of burnout in intensivist doctors who work in adult, pediatric and neonatal intensive care units in five capitals representative of the Brazilian geographic regions with a substantial sample of these professionals.

## METHODS

A descriptive epidemiological study was performed with doctors working in ICUs who agreed to participate in the study. The sample was random and stratified by conglomerate through a lottery of 60 ICUs registered with the *Associação de Medicina Intensiva Brasileira* (AMIB) in five capitals representative of the Brazilian geographic regions (six in Belem (PA) in the northern region, six in Goiania (GO) in the central-west region, 10 in Porto Alegre (RS) in the southern region, 12 in Salvador (BA) in the northeastern region, and 26 in Sao Paulo (SP) in the southeastern region). Ten doctors were randomly selected from every ICU that agreed to collaborate with the study, for a total of 600 workers. The study was approved by the Ethics Committee at the *Universidade Estadual de Feira de Santana* (CEP-UEFS) by Protocol CAAE-21500313.4.0000.0053. The data collection was performed from December 2013 to August 2014. All those who participated in the survey signed informed consent statements.

For data collection, we used a self-administered questionnaire that was anonymous and composed of nine blocks of questions as follows: general identification; general information about the work; psychosocial characteristics of the work; burnout syndrome; quality of life; ability to work; aspects related to health; life habits; and occupational stress factors in the ICU. The questionnaire and informed consent statements were forwarded to workers through the coordinators of the ICU accompanied by a letter of presentation and an explanation of the study. The questionnaires were returned directly to the AMIB in sealed envelopes through correspondence with postage paid to ensure secrecy and confidentiality.

The collected data were organized from criteria established by the instruments used, and the results are presented in tables. The Maslach Burnout Inventory (MBI) was used to identify burnout. The MBI comprises 22 statements concerning feelings and attitudes that compose its dimensions through three 7-point scales (zero to 6) that independently describe each of the dimensions. Emotional exhaustion is evaluated by nine items, depersonalization by five and inefficacy by eight. The cutoff points referenced by the author of the scale were used.^(15)^

For emotional exhaustion, a score ≥ 27 indicates a high level, 17 to 26 indicates a moderate level, and < 16 indicates a low level. For depersonalization, scores ≥ 13 indicate a high level, 7 to 12 indicate a moderate level, and less than 6 indicate a low level. The inefficiency scores trend in the opposite direction, with scores of zero to 31 indicating a high level, 32 to 38 a moderate level and ≥ 39 a low level.^(15)^

Because there is no consensus in the literature for the interpretation of the MBI scale, the results are described according to the signalized criteria of Tucanduva et al.^(16)^ by the presence of three dimensions at a high level or at least one dimension at a high level.

With respect to stress factors in the ICU, 14 factors were presented. The questionnaire requested that the doctor assess the intensity with which the factor stressed him or her on a scale of zero to 3. The answers zero and 1 were grouped as factors that caused no or very little stress, whereas responses between 2 and 3 indicated factors that caused moderate or great stress.

Two databases were constructed using the EpiData program to avoid possible data entry errors. After this step, the data were exported to the Statistical Package for Social Science (SPSS) program. Descriptive analysis of the data was performed based on the calculation of absolute and relative frequencies of categorical variables and means and standard deviations of numeric variables.

## RESULTS

One hundred and eighty intensivists participated in the study (24 from Belem, 18 from Goiania, 28 from Porto Alegre, 65 from Salvador and 45 from Sao Paulo). Of these, 127 (70.6%) worked in an adult ICU, 22 (12.2%) in a pediatric ICU and 31 (17.2%) in a neonatal ICU. This amount represented only 30% of the initially eligible doctors. The losses were caused by the difficulty of accessing the doctors, non-acceptance by the administration at many hospitals, whose ICU were randomly selected to collaborate with the study, direct refusal by the doctors, or inaccuracy in the list of doctors who worked in the ICU. Therefore, the planned random sample became a convenience sample.

Among the intensivists studied, 54.4% were female. This percentage increased when considering the intensivists working in pediatrics or neonatology (86.8%) and decreased for intensivists working with adults (40.9%). The majority (40.2%) were at least 35 years of age, with an average age of 39 ± 8.1 years. Regarding marital situation, 53.6% were married, 30.2% were single, and 53.6% had children. Regarding the highest degree, the majority (63.4%) had specialization, followed by a Masters (19.4%) and a PhD (17.2%). The title of intensive care specialist was held by 46.1% of the intensivists, and 89.4% were residents (Table 1). This percentage increased when considering the doctors who attended adult patients (54.3%) and decreased when considering pediatricians and neonatologists (32.0%). Regarding net monthly income, most (55.4%) had incomes in the range of R$ 10,001.00 to R$ 20,000.00, but it should be stressed that 31.1% of the doctors who attended adults received more than R$ 20,001.00.

**Table 1:** Personal characteristics

	ICU adult N (%)*	ICU pediatric/neonatal N (%)*	Total N (%)*
Female	52 (40.9)	46 (86. 8)	98 (54. 4)*
Age range (years)			
≤ 35	50 (39.4)	22 (42.3)	72 (40.2)
36-45	46 (36.2)	20 (38.5)	66 (36.9)
> 45	31 (24.4)	10 (19.2)	41 (22.9)
Total	127 (100.0)	52 (100.0)	179 (100.0)*
Relationship status			
Single	36 (28.6)	18 (34.0)	54 (30.2)
Married	68 (53.9)	28 (52.8)	96 (53.6)
Consensual union	18 (14.3)	4 (7.5)	22 (12.3)
Widowed	0 (0.0)	1 (1.9)	1 (0.5)
Divorced/separated	4 (3.2)	2 (3.8)	6 (3.4)
Total	126 (100.0)	53 (100.0)	179 (100.0)*
Children	67 (52.8)	29 (55.8)	96 (53.6)*
Highest degree			
Specialization	44 (61.1)	15 (71.4)	59 (63.4)
Masters	14 (19.4)	4 (19.0)	18 (19.4)
PhD	14 (19.4)	2 (9.5)	16 (17.2)
Total	72 (100.0)	21 (100.0)	93 (100.0)*
Title of specialist	93 (71.0)	38 (29.0)	131 (100.0)*
In intensive therapy	43 (46.2)	6 (15.8)	49 (37.4)
In another area	24 (25.8)	24 (63.2)	48 (36.6)
In both	26 (28.0)	8 (21.0)	34 (26.0)
Residency	111 (87.4)	50 (94.3)	161 (89.4)*
Net monthly income (R$)			
< 10,000.00	16 (13.2)	18 (34.0)	34 (19.5)
10,001.00 - 20,000.00	68 (55.7)	29 (54.7)	97 (55.4)
> 20,001.00	38 (31.1)	6 (11.3)	44 (25.1)
Total	122 (100.0)	53 (100.0)	175 (100.0)*

ICU - intensive care unit.

* Valid responses.

With respect to the time working in the ICU, most (55.7%) had up to 10 years of experience, but a significant percentage (28.4%) had more than 16 years of experience. Regarding the hourly workload in the ICU, most (39.8%) worked less than or up to 24 hours a week, although many reported working in the range of 25 to 40 hours (20.4%) and 41 to 60 hours (26.9%).

Most doctors (50.3%) had a total weekly workload in the range of 49 to 72 hours, but 24.2% of those who attended adult patients worked more than 73 hours a week. The most frequent type of employment was salaried private sector employee (36.6%), followed by salaried public sector employee (25.7%) and legal person (20.6%) (Table 2).

**Table 2:** Functional characteristics

	ICU adult N (%)*	ICU pediatric/neonatal N (%)*	Total N (%)*
Time working in ICU (years)			
0 - 5	27 (21.6)	18 (35.3)	45 (25.6)
6 - 10	40 (32.0)	13 (25.5)	53 (30.1)
11 - 15	21 (16.8)	7 (13.7)	28 (15.9)
> 16	37 (29.6)	13 (25.5)	50 (28.4)
Total	125 (100.0)	51 (100.0)	176 (100.0)*
Hourly workload in ICU			
< 24	44 (36.7)	24 (47.1)	68 (39.8)
25 - 40	24 (20.0)	11 (21.6)	35 (20.4)
41 - 60	38 (31.7)	8 (15.7)	46 (26.9)
> 61	14 (11.7)	8 (15.7)	22 (12.9)
Total	120 (100.0)	51 (100.0)	171 (100.0)*
Load weekly total hourly work			
≤ 48	30 (24.2)	21 (39.6)	51 (28.8)
49 - 72	64 (51.6)	25 (47.2)	89 (50.3)
≥ 73	30 (24.2)	7 (13.2)	37 (20.9)
Total	124 (100.0)	53 (100.0)	177 (100.0)*
Institutional employment			
Partner	8 (4.4)	4 (5.3)	12 (4.7)
Legal person	39 (21.4)	14 (18.6)	53 (20.6)
Salaried private	62 (34.1)	32 (42.7)	94 (36.6)
Contract temporary private	5 (2.8)	2 (2.7)	7 (2.7)
Cooperative	2 (1.1)	0 (0.0)	2 (0.8)
Salaried public	49 (26.9)	17 (22.7)	66 (25.7)
Contract temporary public	4 (2.2)	4 (5.3)	8 (3.1)
Service provider	13 (7.1)	2 (2.7)	15 (5.8)
Total	182 (100.0)	75 (100.0)	257 (100.0)*

ICU - intensive care unit.

* Valid responses.

The factors that most stressed professionals are presented as simple frequencies in table 3, organized from most stressful to least stressful. Some of the most frequent factors were related to the direct relationship with users of the services of doctors, with special emphasis on dealing with the suffering of relatives (67.8%), little time to deal with the emotional needs of patients (52.8%), the possibility of complications in patient care (51.4%), and the amount of patients per professional (48.6%). Others reported stressors concerning the structure and the functioning of the ICU, such as excessive noise (56.5%), administrative problems (49.7%), a lack of material resources (47.4%), and an obligation to deal with issues simultaneously (47.2%). Next were the factors that called attention to the work in interdependence with other professionals, such as the commitment of the team (43.7%). One of the factors not considered stressful by the doctors studied was dealing with suffering and death.

**Table 3:** Occupational stress factors in the intensive therapy unit

	ICU adult N (%)	ICU pediatric/neonatal N (%)	Total N (%)
Dealing with the suffering of relatives	75 (60.0)	45 (86.5)	120 (67.8)
Excessive noise	79 (63.2)	21 (40.4)	100 (56.5)
Little time to deal with the emotional needs of patients	61 (48.8)	32 (62.7)	93 (52.8)
Possibility of complications in patient care	57 (45.6)	34 (65.4)	91 (51.4)
Administrative problems	64 (51.2)	24 (46.2)	88 (49.7)
Number of patients per professional	63 (50.4)	23 (44.2)	86 (48.6)
Lack of material resources	55 (44.4)	28 (54.9)	83 (47.4)
Obligation to handle many concurrent issues	54 (43.2)	29 (56.9)	83 (47.2)
Team commitment	57 (46.3)	19 (37.3)	76 (43.7)
Accelerated pace of activities	53 (42.4)	23 (44.2)	76 (42.9)
Terminal patient care	42 (33.6)	28 (53.8)	70 (39.5)
Pressure to discharge patients	44 (35.2)	22 (42.3)	66 (37.3)
Relationship with the team	22 (17.6)	11 (21.2)	33 (18.3)

ICU - intensive care unit.

Regarding burnout, there was a prevalence of high scores in at least one of the three dimensions of the MBI for 56.6% for the doctors who attended children/newborns and 63.8% of the doctors who worked in adult ICUs. Of the three dimensions, high scores were observed in 7.1% of the doctors who worked in the adult ICUs and who were not identified as pediatricians/neonatologists. When we analyzed each of the three dimensions separately, high scores were present in 50.6% for emotional exhaustion, 26.1% for depersonalization and 15.0% for inefficacy (Table 4). Of the doctors who presented a high score in only one dimension, 27.8% (50) had a high level only in emotional exhaustion, 5.0% (9) only in depersonalization, and 3.9% (7) only in inefficacy.

**Table 4:** Occurrence of burnout syndrome in the three dimensions

	ICU adult N (%)	ICU pediatric/neonatal N (%)	Total N (%)
Emotional exhaustion			
Low	27 (21.3)	8 (15.1)	35 (19.4)
Moderate	34 (26.8)	20 (37.7)	54 (30.0)
High	66 (51.9)	25 (47.2)	91 (50.6)
Depersonalization			
Low	64 (50.4)	36 (67.9)	100 (55.6)
Moderate	24 (18.9)	9 (17.0)	33 (18.3)
High	39 (30.7)	8 (15.1)	47 (26.1)
Inefficacy			
Low	70 (55.1)	34 (64.2)	104 (57.8)
Moderate	33 (26.0)	16 (30.1)	49 (27.2)
High	24 (18.9)	3 (5.7)	27 (15.0)
High level in burnout dimensions			
In any dimension	46 (36.2)	23 (43.4)	69 (38.3)
In one dimension	42 (33.1)	24 (45.3)	66 (36.7)
Emotional exhaustion	30 (23.7)	20 (37.7)	50 (27.8)
Depersonalization	6 (4.7)	3 (5.7)	9 (5.0)
Inefficacy	6 (4.7)	1 (1.9)	7 (3.9)
In two dimensions	30 (23.6)	6 (11.3)	36 (20.0)
Emotional exhaustion + depersonalization	21 (16.5)	4 (7.5)	25 (13.9)
Emotional exhaustion + inefficacy	6 (4.7)	1 (1.9)	7 (3.9)
Depersonalization + inefficacy	3 (2.4)	1 (1.9)	4 (2.2)
In three dimensions	9 (7.1)	0 (0.0)	9 (5.0)
Total	127 (100.0)	53 (100.0)	180 (100.0)

ICU - intensive care unit.

## DISCUSSION

The results of this study present an intensivist medical profile that is mostly young, female, married, with children, with up to 10 years of work experience in the ICU, a high weekly workload, and a monthly income of up to R$ 20,000.00. The most common employment type was salaried (private/public). Most doctors had a medical residency and specialization as the highest degree. Less than half of the doctors held the title of intensive care specialist. This profile changes a little when considering doctors who worked in the adult ICUs, who were male and mostly held the title of intensive care specialist.

The prevalence of burnout in this study based on a high score in at least one dimension was 63.8% for doctors who worked in adult ICUs and 56.6% for doctors who worked in pediatric and neonatal ICUs. When considering high scores in the three dimensions simultaneously, burnout was only observed in doctors who worked in the adult ICUs (7.1%). When analyzed separately, the dimension most affected was emotional exhaustion, which is considered a reaction to the demands of the work; in this case, this exhaustion can be translated as both physical and emotional overload. Depersonalization was the second most affected dimension, followed by inefficacy. When we analyzed the occurrence of high levels in two dimensions simultaneously, the combination between emotional exhaustion and depersonalization was emphasized, which in this case had a prevalence of 13.9%.

Three of the four stressors referred to by more than half of the intensivist doctors were related to the relationship with users of these professional services (dealing with suffering of the family, little time to deal with the emotional needs of patients, and the possibility of complications in patient care).

The profile of the professionals analyzed in the present sample was similar to some previous studies. When considering all doctors, this study was similar to more recent studies,^(9,12,17-19)^ that found a predominance of women (between 55 and 76%). However, our results differed from Embriaco et al.^(10)^ and Tironi et al.,^(1)^ who found that only 28% of the doctors were women. These data are explained by separating the doctors by ICU type because the latter two studies did not include pediatric and neonatal ICU, which had a greater percentage of women. Even so, there was an increase in the number of women in the adult ICU, which could indicate an ongoing change in the profile due to the inclusion of women in several medical specialties.^(20)^

Regarding age, marital status and children, the data from this study are similar to other studies^(1,9,10,12,17-19)^ that observed an average age between 34 and 44 years, a percentage of married doctors between 52% and 73% and a percentage of intensivists with children between 46% and 77%. The low rate of *sensu stricto* courses suggests that these professionals prepare much more for practical applications than for teaching and research. Regarding the title of specialist, our results resemble those of Barbosa et al.,^(9)^ who found a percentage of 46.3%, and differ from Tironi et al.,^(1)^ who found a lower percentage (27.0%).

The burnout values found in this study agree with the values reported in the literature^(1,9)^ (between 63.4% and 70.14%) and are higher than the values reported in some studies^(18,19)^ (41.0% and 50.0%). Among the dimensions, emotional exhaustion had the greatest contribution in our results. With respect to the simultaneous occurrence of the emotional exhaustion and depersonalization dimensions, it was not possible to determine which dimension reached a high score first; however, chronic emotional exhaustion caused by interpersonal demands and workload can generate detachment and indifference of the professional for the individuals and families assisted.^(21)^

The high prevalence of burnout, especially when associated with the emotional exhaustion and depersonalization dimensions, might suggest both work overload under varying types of pressure and an imbalance between technical and interpersonal preparation. We believe that there may be a gap in the psycho-emotional training of intensivists, such that the technical preparation, even when of high quality, may not be sufficient for the intensivists to deal with the day-to-day emotional demands of the ICU. This hypothesis is supported by the finding that three of the four factors considered most stressful concerned the relationship with patients and families.^(7,22)^

Finally, we stress some limitations of our study, such as the high number of losses and refusals (70%), which might have generated selection bias, and the descriptive model that prevented the analysis of cause and effect. However, the sample of 180 respondents is adequate to detect a burnout rate of approximately 60% (based on our results), with a sampling error of 7% and 95% reliability. The results also properly represented doctors who worked in units of five regions of Brazil in ICUs that cared for patients of different ages by analyzing the prevalence of burnout on three dimensions separately and in combination.

Our findings effectively contribute to a greater understanding of this phenomenon and assist with organizational policies for the promotion and protection of health in this professional category. This scenario appears unfavorable to doctors who work in the ICU and can affect the quality of care provided to health service users. The results also suggest the lack of psycho-emotional preparation among these professionals, which can indicate the need to rethink medical training in intensive care.

## CONCLUSION

Our study showed that burnout syndrome in intensivist doctors was prevalent when considering the criterion of a high level in at least one of the dimensions evaluated and had a lower prevalence when considering high levels in all dimensions. The emotional exhaustion dimension contributed most to the result, which indicates a need to review the working conditions of these professionals, who feel required to perform beyond their resources.
